# Collectanea Medica

**Published:** 1812-02

**Authors:** 


### COLLECTANEA MEDICA,

CONSISTING OF

### ANECDOTES, FACTS, EXTRACTS, ILLUSTRATIONS, QUERIES, SUGGESTIONS, &c.

RELATING TO THE

*History or the Art of Medicine, and the Auxiliary Sciences.*

### *Veracity in reporting Facts.*

ALL who write cases in medicine would do well to weigh attentively, when they begin to put pen to paper, the following words of Judge Forster:—"Imperfect reports of facts and circumstances, especially in cases where every circumstance weigheth something, are the bane of all science."—*Report and Discourses on the Crown Law*, p. 294.

### *Extraordinary Success in treating the Natural Small-pox.*

We are told by Isaac Massey, the apothecary to Christ's Hospital, who was a redoubtable opponent of inoculation, that Sir Hans Sloane attended *some hundreds* of the children belonging to that establishment in the natural small-pox, and in the space of eight years lost *only one* patient. This, if true, is an instance of success in treating that destructive disease, unparaleled in the annals of medicine.

### *Physicians visiting in Carriages, and increasing their Fee to a Guinea.*

From the following passages in the "Lex Talionis, or a short Reply to Dr. Merrett's Book, written against the Apothecaries," published in 1670, it appears that it was then becoming customary for physicians to make their visits in a carriage, and that they expected a double fee (viz. two angels) on such an occasion:—"For now there must be the little coach*, and two horses, which in these days are very usual appendices to the doctor in physic; and, being thus attended, half a piece, their usual fee, is but ill taken, and popped perhaps into the left pocket, and possibly may cause the patient to send to his worship (before he will come again) to the hazard of another angel."

Before this, physicians of much practice used to visit their patients on horseback: Dr. Simeon Fox and Dr. Argent are said to have been the last presidents of the College who visited their patients in this manner.—*See Aikin's Biographical Memoirs of Medicine*, p. 276.

---

* Query.—Does this mean a chariot? *quasi* a little, or half, coach? the *carrosse coupé* of the French?

 4. State

*State of Surgery in Scotland in* 1585.

Before the commencement of the eighteenth century, every thing connected with the healing art in the Scottish capital was wretched in the extreme. Barbers and surgeons were one and the same profession, who exclusively practised as a craft the dressing of wounds, shaving of beards, and making and selling whisky, throughout the *gude town.*

When the surgeons of Edinburgh were, in 1505, incorporated under the denomination of Surgeons and Barbers, it was required of them to be able *to read and write!* "to knaw anatomie, nature and complexioun of everie member of humanis bodie; and lykwayes to knaw all vaynes of the samyn, that he may make flewbothomea in dew time," together with a perfect knowledge of shaving beards. These were all the qualifications that seemed necessary to the art of surgery at the beginning of the sixteenth century. The practice of physic was, if possible, in a more deplorable state.—*Campbell's Journey from Edinburgh to the Highlands, &c.*

*Popular Treatises on Diseases and Medicines.*

Andrew Borde, physician to Henry VIII. from whom the name of "Merry Andrew" is said to have been derived, seems to have been afraid that popular treatises on medicine might do harm, though he published such himself, for he says in his Breviary of Health, "and where that I am very briefe in shewing brief medicines for one sicknes, I do it for ij causes. The fyrst cause is ẏ the archane science of phisicke should not be too manifest and open, for then the eximious science should fall into great detriment, and doctours the which hath studied ẏ facultie should not be regarded so wel as they are. Secondarily, if I shold write al my mynd, euery bongler wold practise phisicke vpon my booke, wherefore I do omit and leve out many things, relinquishing that I have omitted to doctours of high judgement, of whom I shall be shent for part of these thinges, that I have written in this booke." (1575.)

*Physicians and Apothecaries in partnership.*

It is said, that some physicians and surgeons of the present day, direct their patients to particular houses, where alone they alledge, that their prescriptions can be properly prepared: this is no new custom. In the "Accomplisht Physician" (1670) we are told, "Physicians all or most, being tyed to particular apothecaries, prescribe their bills in terms so obscure, that they force all chance patients to repair to their own apothecaries, pretending a particular secret, which only they have the key to unlock; whereas, in effect, its no other than the commonest of medicines disguised under an an unusual name, on design to direct you to an apothecary, between whom and the physician, there is a private compact of going snips."

*Sydenham's Knowledge of the Latin Language.*

It was the custom among physicians, at the beginning of the seventeenth century, to correspond with each other in Latin. The late Hemy Palmer, esq. of Queen-street, Berkeley-square, was possessed of a large collection of Latin letters, addressed to his maternal grandfather Doctor Hamey, by the most eminent physicians, his cotemporaries. Among these were several in very elegant Latinity, by the celebrated Sydenham; an irrefragable proof of his competency to write in that language, which some writers have much questioned.

It is to be feared that these letters are irrecoverably lost. What a prize they would prove to a modern collector!

*Physicians compelled to temporise.*

The influence of nurses and gossips in the management of infants, was much greater forty or fifty years ago, than it is at present. The great ornament of his profession, Dr. William Hunter, acknowledged that he was obliged to give way to it. Talking with a physician, who was just commencing practice in London as an accoucheur, on the merits of Dr. Cadogan's "Essay upon Nursing, and the Management of Children, from their Birth to three Years of Age," the latter enlarged upon the soundness of Dr. Cadogan's doctrine, and expressed his determination to follow it up in practice to the utmost of his power. "Ah sir," said Dr. Hunter, "what Dr. Cadogan says, is very true, and very rational, but you will find yourself obliged to temporise. Dr. Cadogan's system will not do in chamber practice."

*Early Practitioners of Midwifery in England.*

Physicians appear to have been among the earliest accoucheurs in England. Dr. George Owen in 1588; Dr. Percival Willughby in 1640; Dr. William Harvey in 1653; Dr. Hugh Chamberlen in 1672; are celebrated as practitioners of midwifery. In France this branch of practice was first exercised by, and long confined to, surgeons only. Alphonse le Roy, in a letter to the late Dr. Leake, dated June 15, 1778, says, that he was the first physician in France who had devoted himself to that branch of practice.

*Accoucheurs.*

In London, most of the physicians who practise midwifery confine themselves to that branch of the profession, and expect little employment in any other branch. It would appear odd for a physician of the present day to designate himself by the title of *Physician Extraordinary to his Royal*

*Highness*

*Highness the Prince of Wales, and* MAN-MIDWIFE. Such, however, was the designation which Dr. William Douglas, the rude opponent of Smellie, chose to adopt.

*Purchasers of De Bruyn's Secret.*

Two physicians of Amsterdam, having been led to believe that De Bruyn's secret, if made public, might prove of very extensive benefit to lying-in-women, from motives of the purest benevolence, purchased the secret from him for 5000 livres. This secret proved to be the vectis or lever, which these physicians immediately published to the world, with all necessary directions for using it. Their names were Vischer and Vandepol. Vandepol afterwards, for what reason is not known, quitted his own country and retired to Canterbury, where he lived more than twenty years, under the assumed name of Dawkins. He died on the 7th of March, 1784, aged 75 years, very much regretted by the poor of that place, to whom he was a liberal benefactor.

*Measles inoculated!*

About twenty years ago, a child who had been inoculated for the small-pox was attacked with the measles, while under the variolous eruption. As it was not suspected that the measles would be communicated by the inoculation, matter was taken from one of the pustules, and inserted into the arm of another child; and this child likewise had the measles and small-pox conjointly. Was this an accidental coincidence of the two diseases? or was the morbillous infection communicated together with the variolous by inoculation? The children were related, but did not live together.

*Permanency of morbid Contagion.*

The dried scales of the vaccine pock have been known to communicate the disease at the distance of two years from the period of their dropping off the arm. Small-pox matter, taken upon a thread by a surgeon in the country, was employed for inoculation by his successors nine years after his death, and communicated the disease in nineteen out of twenty instances.

---

*On the Diseases of the Icelanders.—By* DR. HENRY HOLLAND.

The poverty of the Icelanders, and the dispersion of their small community over so vast an extent of country, render it almost impossible that medical practitioners should obtain an independent subsistence in the island. To obviate, as far as possible, this evil, a small medical establishment is provided at the public expence; consisting of a superintendant physician, who has the title of Landphysicus, an apothecary, and five subordinate medical men, who are stationed in different parts of the island. The physician and apothecary are are settled in the vicinity of Reikiavik; where a house somewhat superior in size and accommodation to the common class of Icelandic habitations, is provided for their reception. Independently of this provision, and the use of some land annexed to the house, the landphysicus has an annual salary of 600 rix-dollars, with the liberty to avail himself of the profits of any practice which his situation may afford. The present possessor of the office is Dr. Klog, a native of Iceland, but educated at Copenhagen. Of the country practitioners, one is stationed on the southern coast of the island, another on the eastern coast, a third on the northern, and two in the western province. The reader will readily conceive how entirely destitute of medical assistance many parts of the country must be, when it is mentioned that some of these districts, subject to the care of a single individual, extend nearly 200 miles along the coast, with a breadth varying from ten to thirty miles. We had the opportunity, while in Iceland, of seeing two of the country practitioners; both very respectable men, and well informed in their profession. One of them, Mr. Paulson, has already been noticed, as possessing a more extensive knowledge of natural history than any of his countrymen.

With the exception of three hospitals, in which a few incurable lepers receive gratuitous support, no medical institution exists on the island. These hospitals are maintained at the public expence, and in a method worthy of being noticed, from its singularity. On a certain specified day, at that time of the year when the fishery on the coast is most abundant and successful, every fishing-boat in the island is required to contribute one man's share of the capture that has been made; a provision is added to the law, that, if the number of fish taken by any boat on this day does not afford a share of five to each fisherman, the contribution to the hospitals shall be delayed until the next time, when the produce of a day's fishing equals or exceeds this amount.

In speaking of the diseases of Iceland, it will be necessary to allude only to those which furnish any facts peculiar and interesting, or which are more especially connected with the climate and mode of living among the inhabitants.

The diet of the Icelanders consists almost solely of animal food; of which fish, either fresh or dried, forms by far the largest proportion. During the summer, they have milk and butter in considerable abundance; but of bread, and every other vegetable food, there is the utmost scarcity, and, among the lower classes, an almost entire privation. The want of cleanliness in the personal and domestic habits of the people, has been frequently alluded to; it is an evil incident to their situation, the removal of which could probably only be accomplished by by the sacrifice of other habits, still more essential to their comfortable existence. As an effect of these circumstances in the mode of life of the Icelanders, cutaneous diseases, arising from a cachectic state of the body, are exceedingly frequent among them, and appear under some of their worst forms. Scurvy and leprosy are common in the island, occurring especially in the districts of Gutbringe and Snæfell Syssols, and on other parts of the western coast, where the inhabitants depend chiefly upon fishing, and where the pastures are inferior in extent and produce. The scurvy *(kreppusot)*, as it appears in Iceland, presents no remarkable peculiarity of symptoms. The disease is observed to occur with greater frequency at those periods when there has been a deficiency of food among the inhabitants, or when the snow and frost of the winter succeed immediately to a wet autumnal season. For its cure a vegetable diet is employed, in as far as the circumstances of the Icelanders will allow of such means. Fruits of every kind are altogether wanting to them ; but some advantage is derived from the employment of the cochlearia *(officinalis et darica)*, of the *trifolium repens*, of the berries and tops of the *juniperus communis*, and of the *sedum acre ;* plants which are indigenous in the island.

The leprosy of the Icelanders *(likthra*, *holdsveike*, or *spitelska*,*)* exhibits, in many instances, all the essential characters of the genuine elephantiasis, or lepra arabum ; and is a disease of the most formidable and distressing kind. Indolent tumors of the face and limbs are generally among the first symptoms of the complaint, attended by swellings of the salivary, inguinal, and axillary, glands. The nostrils, ears, and lips, are progressively affected with swelling and deformity. The skin over the whole, or different parts of the body, becomes thick and hard, sometimes exhibiting a shining or unctuous surface, sometimes one rough and scabrous ; which, at a more advanced period of the disease, displays numerous cracks or fissures. The senses are usually much enfeebled, and anæsthesia of the extremities generally occurs. The voice assumes a peculiar hoarseness and nasal tone, frequently with swelling of the tonsils, but without any hindrance of deglutition, until the disease has made a great progress in the habit of the patient: the breath and perspired matter are extremely fœtid ; and the hairs and nails frequently fall off. The tumors in the different parts of the body gradually pass into malignant ulcers, which discharge an acrid unhealthy matter. In this state the patient often lingers during a long life ; or, when the disease has a more speedy termination, all the symptoms are rapidly aggravated, gravated, and he is carried off in a state of extreme debility and wretchedness.*

When it is considered how frequently unsuccessful the treatment of this disease is in more auspicious regions, it will not excite surprise that in Iceland the attempt at cure should generally be unavailing, where, from the situation of the patient, medical assistance can be obtained. Laxatives, diaphoretics, and issues, or sometimes even venesection, are employed in the earlier stages, or with a prophylactic view. The indigenous plants which the natives employ as remedies, are, the juniper, the *vaccinium myrtillus*, the *rhodiola-rosea*, and the *dryas octopetala*; the latter of which, particularly, grows in great abundance on the island. These remedies, however, appear to be of little avail in relieving any of the urgent symptoms of the disease.

It does not appear that any distinct record exists in Iceland of the first appearance of the leprosy in this country. The Chevalier Bach, in his letter to Dr. Van Troil on the subject, thinks it probable that the disease was brought into Iceland from Asia or the South of Europe, at the time of the crusades; in which he asserts, that these Icelanders bore a part with the other nations of Europe.† From the Ecclesiastical History of Iceland, it appears that the latter statement is not well founded; but though not participating in the holy wars, the Icelanders had at this period an intimate connection with the European continent; and the disease of which we are speaking, when once introduced, would readily be kept up, partly by its contagious character, principally perhaps by the food and personal habits of the people. In the rest of Europe it has gradually disappeared, in consequence of the progressive improvement in the modes of living among every class of society.

The ravages committed by the small-pox in Iceland have been so much as to render this disease important even in the political history of the island. Introduced from the continent at different periods, and these in general distant from each other, it has spread rapidly, and under its most virulent form; producing effects almost unexampled in the history of this dreadful disease. The most remarkable instance of this kind occurred in 1707; during which year the mortality amounted, according to the most accurate estimate, to about 16,000 souls; more than a fourth part of the whole

* This Icelandic disease is said to be the same with the Elephantiasis of Cullen, the E. legitima of Sauvage; and agrees very exactly with that described by Dr. T. Heberden (Lond. Med. Trans. vol. i.) as occurring in the island of Madeira.

† Van Troil's Letters on Iceland.

 population population at that period. Several similar instances are recorded in the history of Iceland, though none attended with effects so extremely disastrous. A few years ago the vaccine matter was introduced into this island from Denmark; but, owing to the smallness of the population, and its dispersion over so wide a surface, this was soon lost again; and, at the time of our arrival (in May 1810), we found the practice of inoculation (vaccination) entirely suspended. In contemplation of this circumstance, we had taken out with us a few vaccine crusts, with the design of recommending the method lately proposed by Mr. Bryce. Almost immediately on our arrival, we inoculated several children at Reikiavit, and afterwards in other parts of the country; and, having a communication with the landphysicus on the subject, we had the satisfaction of knowing, before we returned to Britain, that the vaccine crust had found its way into every part of the island. The adoption of the plan of inoculating from the crusts, will doubtless secure to the inhabitants a permanent continuance of this blessing.

The Icelanders have occasionally suffered much from the measles, as well as from the small-pox; in 1797 six hundred people were carried off by this disease.

Syphilis cannot be said to exist in Iceland. Single cases have sometimes occurred from communication with foreigners; but the disease has always been intercepted before it made any progress in the country.

Psora is an almost universal complaint in Iceland, appearing indiscriminately among all classes of the inhabitants. No discredit is attached to it, nor does it seem that any means of cure are attempted, though the most efficacious remedy is found in so much abundance in the country.

Inflammatory visceral affections are very common among the Icelanders. The variable nature of the climate, and the constant exposure to wet and cold which is incurred in the occupation of fishing, give a strong tendency to pulmonary complaints; and, out of the annual number of deaths in the island, a very large proportion are referable to this cause. This fact was ascertained from the examination of certain statistical registers, which are annually drawn up by the priests of the several parishes, and transmitted to the bishop of Reikiavik. In these pulmonary affections, and especially in cases of phthisis, the *lichen islandicus* is much employed by the natives; and possesses a reputation among them, which the experience of its effects in other countries would scarcely seem to warrant. As a demulcent remedy, however, it probably in some degree alleviates the symptoms, and, and, as an article of diet, in such cases its use may certainly be advantageous.

Inflammatory affections of the abdominal viscera are likewise very common among the Icelanders; chiefly, perhaps, in consequence of the peculiar nature of the diet to which they are accustomed. It is possible also that a disposition may be given to these complaints by the treatment of the children in their early infancy. A mother in Iceland seldom suckles her child; but nourishes it with cows' or sheep's milk, which the infant sucks from a piece of moistened rag, or a sponge. Where, from extreme poverty, or other circumstances, milk cannot be obtained, a little fish or flesh meat, rolled up in cloth and linen, and put into the infant's mouth, is the substitute most commonly employed. The diet of the Icelanders likewise gives much disposition to worms; and the ascarides are observed to be particularly frequent.

The climate and the occupations of the people, particularly that of fishing, render rheumatic affections very common. It is said that gout also occasionally occurs; but it may be doubted if it be not some modification of rheumatism which obtains this name.

Hypochondriasis is a frequent complaint among the natives of Iceland, induced probably by the physical circumstances of their situation, and the long confinement to their habitations, which is necessary during the winter season. Yet the general temperament of the Icelanders does not appear to be a melancholic one, and the vivacity of their manner frequently forms a striking contrast to the wretchedness which their external condition displays.

Besides the diseases which have already been noticed, I had the opportunity, while in Iceland, of seeing cases of epilepsy, hysteria, amenorrhœa, menorrhagia, asthma, icterus, &c. No case of idiopathic fever, either intermittent or continued, occurred to my observation. With respect to intermittents, however, I was informed that they occasionally appear among the inhabitants under a well marked form; an effect, no doubt, of the vast extent of bogs and marshy ground, which are formed even in the most populous districts of the island.

A singular complaint remains to be noticed, the effects of which, though limited to a small spot, are eminently disastrous as far as they extend. This is the disease called *Ginklofe* by the Icelanders; the Tetanus or Trismus neonatorum of medical writers; which invades children at a very early age, and almost invariably proves fatal in its event. It occurs very rarely, if at all, on the mainland of Iceland; but is is confined principally to the group of islands called Westmann-Eyar, situated on the southern coast*. The population of Heimacy does not amount at present to above two hundred souls, and is almost entirely supported by migration from the main land; scarcely a single instance has been known, during the last twenty years, of a child surviving the period of infancy. During a great part of the year, the island is wholly inaccessible in consequence of storms, currents, and the nature of the coast. The inhabitants are therefore left almost solely to their own resources. The chief article of food is the sea fowl, called *fulmar*, which they procure in vast abundance, using the eggs and the flesh of the bird, and salting the latter for their winter food. The destructive effects upon the fishery around these islands by the volcanic eruption of 1783, has deprived the inhabitants of this article of diet. Of vegetable food they have none; and there are only a few cows and sheep on the island.

The distressing consequences of this disease led the Danish government to give an official direction to the landphysicus of Iceland, to visit the Westmann islands, for the purpose of investigating its nature and causes. This gentleman went over to the islands during the summer of 1810, and remained three weeks on the spot. Though he did not himself see a case of the disease, he obtained all the principal facts connected with it from the priests, and those of the inhabitants who had had children. The symptoms of the complaint are briefly these. Very soon after birth, strabismus and rolling of the eyes are observed; subsultus tendinum occurs; and the muscles of the back are often drawn together and stiffened, evidently by incipient spasm. These appearances infallibly denote the approach and event of the disease. Having continued during a period varying from one to seven days after birth, trismus generally comes on, sometimes attended by opisthotonos, which is strictly called the *ginklofe*; occa-

* The Westmann islands consist entirely of lava. The nearest to the coast is about twelve miles distant; and the most remote about twice that distance. Only one of them, called *Heimacy*, is inhabited; and the people are by no means respected by their neighbours on the main land, who represent them as being remarkably indolent and depraved in their habits. Their food consists chiefly of fulmars and puffins (*Procellaria glacialis, et Alca arctica, Linn.*), which are slightly salted and barrelled. This is the principal aliment of the people of St. Kilda, the most remote of the western islands of Scotland. The same peculiar and fatal disease attacks children in both places. Until the great volcanic eruption of 1783 took place, there was abundance of fish around the Westmann islands; but, since that period, the fishing on this coast is reported to be much less productive.

sionally sionally with emprosthotonos, to which the name of *klums* is given by the natives. The trismus presently impedes deglutition, and, the paroxysms becoming more violent, the infant is carried off. When the rare event of a favorable termination occurs, it is portended by a critical diarrhœa, or by an exanthematous eruption, with the evacuation of the meconium.

The following Table, which includes a period of twenty-five years, shews the mortality consequent upon this disease in the Westmann islands; and exhibits the days also upon which death has happened.

| Children. | | Days. | Children. | | Days. |
|---|---|---|---|---|---|
| 1 | lived | 2 | 18 | lived | 9 |
| 3 | | 3 | 10 | | 10 |
| 14 | | 4 | 2 | | 11 |
| 16 | | 5 | 1 | | 12 |
| 22 | | 6 | 1 | | 13 |
| 75 | | 7 | 5 | | 14 |
| 16 | | 8 | 1 | | 21 |

It will be seen, from this Table, that the number of deaths on the 7th day greatly exceed those on any other; and also that they are more frequent on the 14th day, than on the days immediately preceding or succeeding it. From the proportion which these cases of fatal event bear to the whole population of the island, it is probable that few, if any, instances of recovery have occurred, during the period included in the Table. No methods of cure have hitherto been resorted to by the inhabitants.

This disease is well known to prevail in other parts of the world; and has been particularly described as it appears in the West Indies, and in the island of Minorca*. It exists also in Switzerland, and in some of the northern districts of Scotland; especially in the island of St. Kilda, the inhabitants of which, in their diet and mode of life, much resemble the natives of the Westmann islands. The exciting causes are involved in much obscurity. It may be presumed, however, that they must vary considerably, when the disease appears in countries so widely different, with respect to climate and the situation of the inhabitants. Its occurrence in the Westmann islands may reasonably be supposed to have some connection with the extraordinary diet of the natives; and this is the more probable, as it appears that the complaint has been much more frequent since their fishery was de-

* Vid. Hillary, Chisholm, and Clarke, on the Diseases of the West Indies; and Cleghorn's Diseases of Minorca.

stroyed stroyed by the volcanic eruption in 1783. Independently of any effect which the peculiarity of the mother's constitution may have upon her offspring, the practice of giving to the infant a strong and oily animal food almost immediately after birth, will necessarily create irritation in the bowels, and dispose to spasmodic affections. Dr. Klog, in some remarks he gave me on this subject, attributes much to the effects of the sea air, and of a moist atmosphere; but, had these causes any considerable influence, we might expect that the disease would be more frequent in different parts of the world, than is actually found to be the case.

The age which the Icelanders usually attain, presents nothing remarkable in either extreme: from a table of population, it appears that in 1801, when the number of inhabitants was 47,207, there were 41 persons between the ages of 90 and 100; 443 between 80 and 90; and 1698 between 70 and 80. The number of females was 25,371: of males, only 21,746. The longevity of the females exceeds considerably that of the males; owing, no doubt, to their less exposure to the severities of labour, and the hardships of the climate. Of the 41 persons between 90 and 100, 35 were females; of those between 80 and 90, 285 were females, while the number of males was not more than 158. A comparison of facts would probably prove, that the longevity of the Icelanders rather exceeds, than falls short, of the average obtained from the continental nations of Europe.

The Icelanders are in general of a tall stature; arising, however, more from the length of the spine, than of the limbs: the head is of the middle size: the countenance open: the complexion exceedingly fair, and among the women often very florid. The hair is almost invariably of a light colour, and seldom curled. Corpulency is rarely observed among the natives of Iceland.—*Sir George Stuart Mackenzie's Travels.*

---

### *Experiments on Cantharides.—By* M. Rubiquet.

(Ann. de Chimie.)

The principal object of M. Rubiquet in these experiments, was, to ascertain whether the vesicating property, for which these insects are so remarkable, resides in the whole of the animal, or belongs to some peculiar substance capable of being separated by chemical menstrua from the rest. After various attempts more or less successful, Mr. R. discovered the following simple process, by which the vesicating principle may be obtained in a state of perfect purity.

Take any quantity of bruised cantharides and boil them in distilled water, strain off the reddish-brown decoction, and digest the residue two or three times more in boiling water, till till the last decoction comes off nearly colorless. The insoluble residue affords a green tincture with alcohol, which, being gently evaporated, leaves behind a green fluid oil entirely destitute of any vesicating property.

The aqueous decoction being reduced, by evaporation, to the state of a soft extract, is to be repeatedly digested in boiling alcohol as long as any thing is taken up by this menstruum. The insoluble portion is a black matter soluble in water, and incapable of vesicating. The alcoholic solution is of a yellow color, and excessively acrid. This latter is to be reduced to a soft state by gentle evaporation, and the yellow residue is to be digested in cold sulphuric ether, in a well stopped vial, and frequently shaken. In the course of a day or two the ether will have acquired a pale yellow color, when it is poured off from the undissolved portion, and exposed to spontaneous evaporation. By this method there will be deposited a quantity of micaceous-looking scales, fouled with a few small drops of a yellowish fluid. This may be removed by subsequent digestion in cold alcohol, and the micaceous crystallised matter alone will remain. This latter is the pure vesicating principle; the smallest visible particle dissolved in a little oil (in which it is readily soluble) and applied to the skin, will in an hour or two raise a perfect blister.

*Observations.*—The experiments of M. Rubiquet are interesting as far as they go, but by no means complete the chemical history of the vesicating principle. To the medical practitioner they offer a method of obtaining this substance in a state in which it is soluble in oil, and may, therefore, be advantageously employed in stimulating frictions.

---

### *Experiments on Camphoric Acid.* *By* M. Bucholz.

(Journ. de Phys.)

The comparative experiments here detailed, relating to the camphoric and benzoic acids, were undertaken by the author of this paper in consequence of a memoir by Doerfurt, maintaining, that they were only varieties of one and the same acid. The following differences, however, pointed out by M. Bucholz, seem to give the camphoric acid as good a claim to be considered as a peculiar species, as is possessed by many other of the acknowledged vegetable acids.

The *camphoric* acid, by slow cooling, takes the form of plumose crystals, like those of muriated ammonia: to the taste it is very acid, with an after flavour of bitterness. It is soluble in 100 parts of water, at the common temperature; and in 10 or 11 parts of this fluid, when boiling hot, cold alcohol takes up a very little more than an equal weight of

 this this acid, and hot alcohol dissolves 1·5 part. When sublimed, the greater part of the acid is decomposed, an empyreumatic oil, with a very peculiar odour, is formed, together with a liquid acid, and much charcoal: 100 parts of camphoric acid require for perfect saturation 56 parts of carbonated lime, forming a neutral salt, which dissolves in five parts of cold water, and crystallised in round heaps: to the taste it is weakly saline and bitter; and when exposed to heat, it decomposes without fusion, giving out an oil which has the odour of rosemary.

*Benzoic* acid, on the contrary, crystallises in small needles, is slightly acid, saccharine, and pungent to the taste; requires 100 parts of water at the common temperature, and 24 parts of boiling water for its solution; alcohol, when cold, takes up half its weight; and when hot, takes up an equal weight of this acid: when sublimed, it undergoes very little decomposition, and produces no aqueous vapours: 100 parts of benzoic acid, require about 20 of calcareous carbonate for perfect saturation; and the benzoate of lime thus produced, is soluble in 20 parts of cold water, crystallises in brilliant laminæ arrayed like stars; to the taste is sweet, and somewhat earthy; and, when exposed to heat, becomes fluid, and gives out an empyreumatic oil of the flavour of Peruvian balsam, together with benzoic acid in crystals.

